# Underwater superoleophobicity, anti-oil and ultra-broadband enhanced absorption of metallic surfaces produced by a femtosecond laser inspired by fish and chameleons

**DOI:** 10.1038/srep36557

**Published:** 2016-11-07

**Authors:** K. Yin, Y. X. Song, X. R. Dong, C. Wang, J. A. Duan

**Affiliations:** 1 State Key Laboratory of High Performance and Complex Manufacturing, School of Mechanical and Electrical Engineering, Central South University, Changsha 410083, China

## Abstract

Reported here is the bio-inspired and robust function of underwater superoleophobic, anti-oil metallic surfaces with ultra-broadband enhanced optical absorption obtained through femtosecond laser micromachining. Three distinct surface structures are fabricated using a wide variety of processing parameters. Underwater superoleophobic and anti-oil surfaces containing coral-like microstructures with nanoparticles and mount-like microstructures are achieved. These properties of the as-prepared surfaces exhibit good chemical stability when exposed to various types of oils and when immersed in water with a wide range of pH values. Moreover, coral-like microstructures with nanoparticle surfaces show strongly enhanced optical absorption over a broadband wavelength range from 0.2–25 μm. The potential mechanism for the excellent performance of the coral-like microstructures with a nanoparticle surface is also discussed. This multifunctional surface has potential applications in military submarines, amphibious military aircraft and tanks, and underwater anti-oil optical counter-reconnaissance devices.

Interfacial materials with multiple properties offer many opportunities for the design of advanced technologies^1^,^2^,^3^,^4^,^5^. Recently, underwater superoleophobic surfaces have emerged with potential applications in submerged antifouling^6^, oil-water separation^7^,^8^,^9^ and small oil-droplet transportation^10^,^11^,^12^. Taking inspiration from nature is an exciting approach for designing smart and functional materials^5^. For example, fish can retain moisture outside of water, swim freely underwater and maintain clean scales even in oil-polluted water^13^,^14^. Inspired by fish scales, many studies have focused on the fabrication of underwater superoleophobic surfaces using various methods^15^. Zhang *et al.* reported a series of underwater superoleophobic Ni/NiO surfaces with controlled oil adhesion by combining electrodeposition and heating techniques^16^. Cheng *et al.* proposed a self-assembled monolayer technique to modify nanostructured copper substrates to prepare tuneable adhesive underwater superoleophobic surfaces^17^. However, these manufacturing processes are difficult to control, and the reported underwater superoleophobic surfaces only exhibit improved oil wettability, greatly limiting their application to underwater devices such as oil-repellent military submarines and amphibious military aircraft and tanks, which also require optical stealth. Therefore, exploring a simple and effective method for fabricating superoleophobic, anti-oil and ultra-broadband enhanced absorptive metallic surfaces remains an urgent and significant challenge.

In another inspiring natural phenomenon, chameleons can change their skin colour depending on the ambient environment, which helps them avoid danger. Underwater reconnaissance devices and amphibious military aircraft and tanks are detectable using visible and infrared broadband wavelengths, among other techniques. The chameleon inspires us to develop novel strategies to address the need for underwater reconnaissance devices with strong surface absorption over the broadband wavelength range. Recently, femtosecond (fs) lasers have proved to be a promising tool for controllable^18^,^19^,^20^, scalable^21^, highly efficient^22^,^23^ three-dimensional processing^24^,^25^,^26^,^27^,^28^. A.Y. Vorobyev *et al.* used femtosecond-laser surface structuring to change the reflectivity of metals and create so-called “colour metals” and “black metals”, through which high absorption across the visible to ultraviolet range was achieved^29^,^30^,^31^,^32^,^33^. However, those structures’ processing parameters were not discussed in detail, and absorption over the broadband wavelength was not sufficiently high.

In this study, underwater superoleophobicity, anti-oil properties and ultra-broadband enhanced optical absorption on metallic surfaces are achieved by direct femtosecond laser ablation in air. By varying the laser-processing parameters, three surface nano/microstructures including coral-like microstructures with nanoparticles (CMN), mount-like microstructures (MM) and grating-like nanostructures (GN) are generated. The CMN and MM surfaces achieve underwater superoleophobicity and anti-oil properties and exhibit good chemical stability with various oils and in a range of pH values. In addition, the CMN surface shows strongly enhanced absorption over a broadband wavelength range from 0.2–25 μm. A potential mechanism underlying the excellent performance of the CMN surface is also discussed. This multifunctional surface shows potential applications in military submarines, amphibious military aircrafts and tanks, and underwater anti-oil optical counter-reconnaissance devices.

## Results

**Figure 1:**
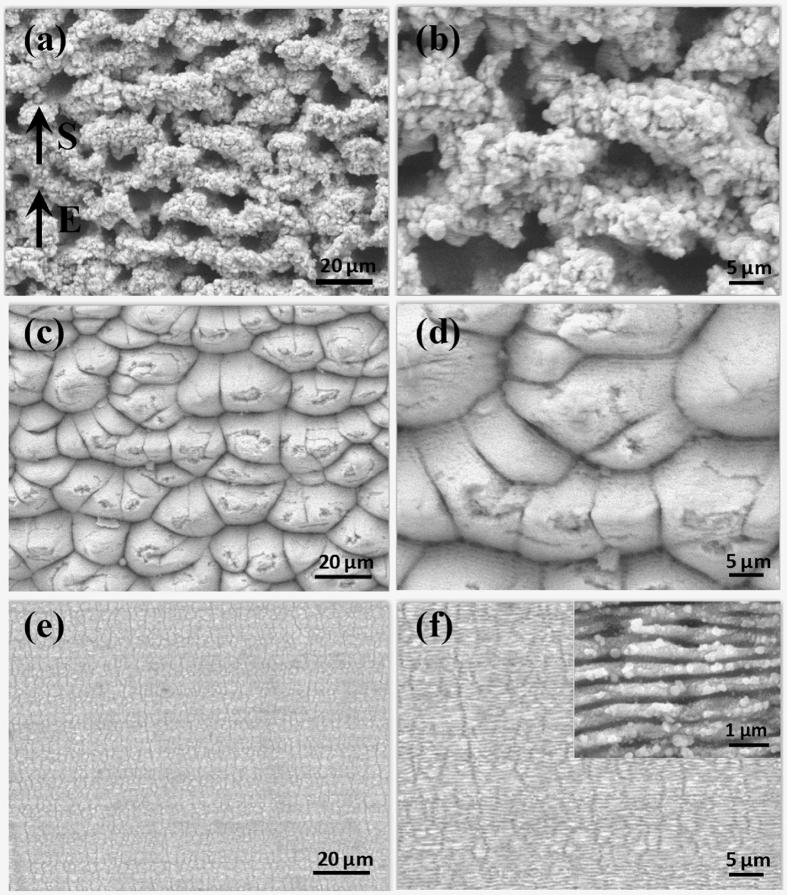
Three surface morphologies of stainless steel plates irradiated by a femtosecond laser with varying laser fluence at a scanning speed of 0.4 mm/s. (**a**,**b**) 80.0 J/cm^2^, (**c**,**d**) 15.0 J/cm^2^, (**e**,**f**) 1.5 J/cm^2^. E is the direction of the laser polarization, while S is the direction of the sample scanning.

Figure 1 shows scanning electron microscope (SEM) images of three surface morphologies of stainless steel plates irradiated by a femtosecond laser at different laser fluences. The scanning speed is 0.4 mm/s. As exhibited in Fig. 1(a), the CMN surface consisting of micro-cavities with random orientations is achieved at a fluence of 80.0 J/cm^2^. The cavities have a diameter of 3–10 μm. As shown in the magnified image of Fig. 1(b), plentiful protrusions are observed on the sidewalls and edges of the cavities, which are considered a conglomeration of nanoparticles ejected from the ablation craters. Meanwhile, MM surfaces can be obtained at a fluence of 15.0 J/cm^2^. As shown in Fig. 1(c,d), the surface contains many micro-mount patterns, some of which are covered by defects and cracks. When the laser fluence is decreased to 1.5 J/cm^2^, the GN surface forms on the metal surface, as depicted in Fig. 1(e,f). The GN has a regular periodic pattern, the orientation of which is perpendicular to both the laser polarization and the scanning direction. The average period is approximately 600 nm.

**Figure 2:**
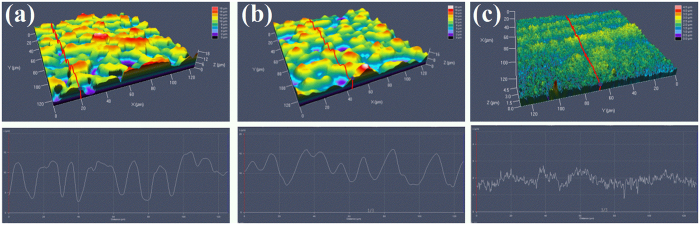
3D confocal microscopy images and profile curves of the three morphologies. (**a**) CMN surface, (**b**) MM surface and (**c**) GN surface.

The 3D profile of the fabricated patterns is characterized using laser confocal microscopy. As shown in Fig. 2(a), 3D coral-like microstructures can be observed on the CMN surface, and the cavity depth is approximately 10 μm. However, according to Fig. 2(b), the average depth of the MM surface from the peak to the valley is only several microns. On the GN surface, the microstructures disappear, leaving only a wavy line with a depth of a few dozen nanometres.

**Figure 3:**
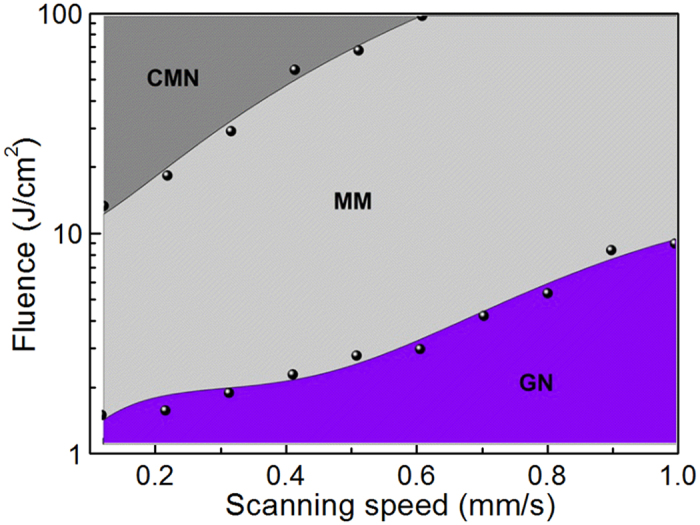
Overview of processing parameters for the formation of the three surface morphologies on stainless steel.

More detailed experiments are also conducted to study the formation of the femtosecond-laser-induced surface structures in terms of laser fluences and scanning speeds, as shown in Fig. 3. Each point is determined by averaging five data points and calculating the standard deviation. For clarity, error bars corresponding to the standard deviation are not shown. All three structures can be formed by varying the processing parameters. At a scanning speed of 0.1–1 mm/s with a relative low fluence, the GN surface can be generated. When the scanning speed or fluence is increased, the MM surface emerges. However, the CMN surface is formed only at high fluence and low scanning speed.

## Discussion

**Figure 4:**
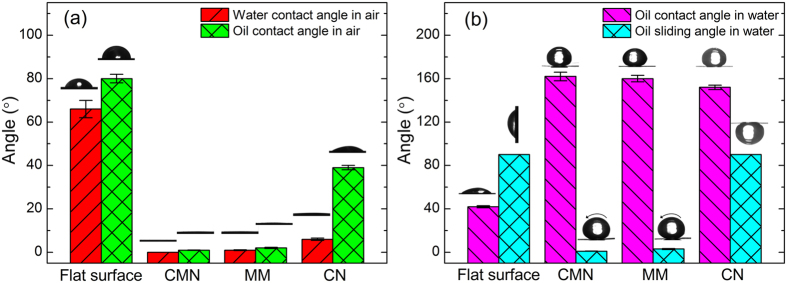
(**a**) Water (6 μL) and oil (6 μL, 1,2-dichloroethane) contact angles on various surfaces in air; (**b**) oil contact and sliding angles on various surfaces in water. Inset images are the profiles of the liquid droplets on the corresponding surfaces.

The wetting properties of the surfaces are measured using a contact-angle system, the results of which are depicted in Fig. 4. According to Fig. 4(a), all surfaces show hydrophilicity and oleophilicity in air. The water contact angles of a control untreated surface and the CMN, MM, and GN surfaces are approximately 66°, 0°, 1°, and 6°, respectively, and the oil contact angles of those surfaces are approximately 80°, 1°, 2°, and 39°, respectively. Interestingly, the oil wettability obviously changes once the as-prepared surfaces are immersed in water. Figure 4(b) shows the oil contact and sliding angles for the four surfaces in water. The flat surface clearly shows underwater oleophilicity and high adhesion, on which an oil droplet can be pinned with a contact angle of 90°. After laser treatment, the oil contact angles of the three treated surfaces are all above 150°, indicating underwater superoleophobicity. However, the oil sliding angles of these surfaces are quite different. The oil droplet can easily roll on the CMN and MM surfaces underwater, with sliding angles of approximately 1° and 3°, respectively, which indicates low oil adhesion on these surfaces. However, oil droplets show high adhesion on the GN surface; an oil droplet can even be pinned when the surface is turned upside down, as shown in Fig. 4(b).

**Figure 5:**
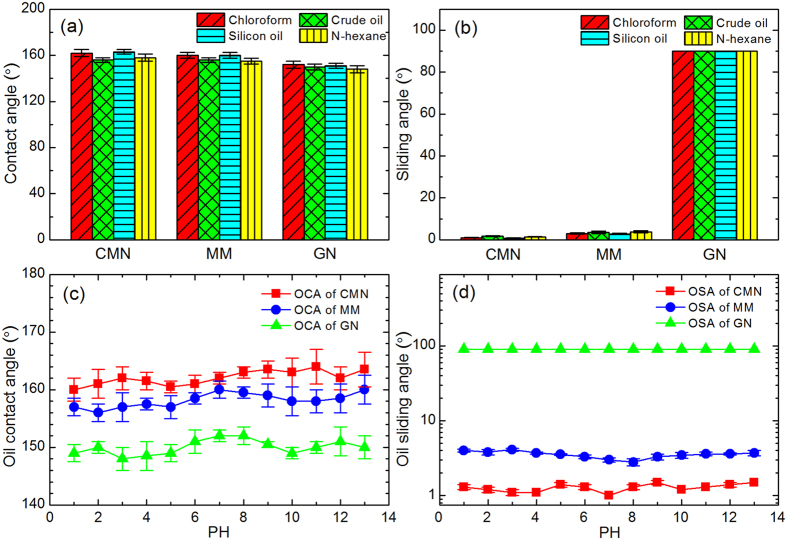
Underwater oil contact angle (**a**) and sliding angles (**b**) for various oils on the as-prepared surfaces. Oil contact angles (**c**) and sliding angles (**d**) on the as-prepared surfaces as a function of aqueous pH.

To investigate the chemical stability of the underwater oil wettability, various oils were dropped on the as-prepared surfaces, which were immersed in neutral water. In addition, surfaces with 1,2-dichloroethane droplets were immersed in water with varying pH. Figure 5(a,b) show the underwater oil contact angle and sliding angles for various oils on the as-prepared surfaces. The surfaces exhibit underwater superoleophobicity with all oils, including chloroform, crude oil, silicon oil and N-hexane, with oil contact angles larger than 148°. The CMN and MM surfaces show low adhesion to all oils, with sliding angles less than 4°. In contrast, the GN surface shows high adhesion; all oil droplets can be pinned with a contact angle of 90°. Figure 5(c,d) show the oil contact angles and sliding angles as a function of the pH of the immersion bath. The OCA and OSA of the as-prepared surfaces are stable at pH values from 1 to 13. These results indicate that the surface properties have good chemical stability and could be applied in harsh water or oil environments. The long-term stability, mechanical durability and scalability of the CMN surface are tested, and all measured values are favourable (see Supplementary Figs S1–S3).

**Figure 6:**
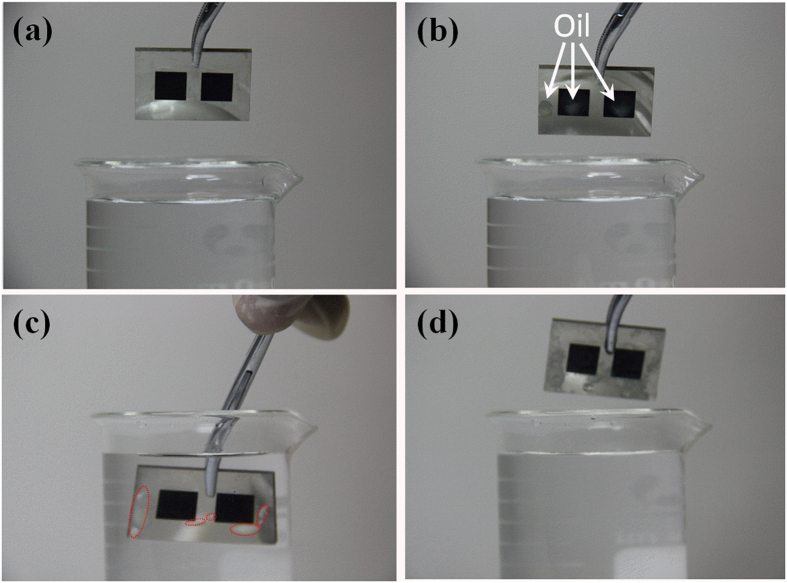
Self-cleaning anti-oil ability of CMN (left) and MM (right) surfaces and the untreated surface.

The CMN and MM surfaces with underwater superoleophobicity and low adhesion have potential applications for self-cleaning and anti-oil devices. Figure 6 exhibits the self-cleaning anti-oil ability of CMN and MM surfaces and an untreated metallic surface. As presented in Fig. 6(a), CMN (left) and MM (right) surfaces (8 mm × 8 mm) are fabricated on the stainless steel plate. Three milky coloured droplets of 1,2-dichloroethane (the milky appearance of this oil is due to the addition of an oily stain) are dropped onto the two treated surfaces and the untreated surface. The samples are fully immersed into water to examine the self-cleaning ability. Interestingly, the oils spontaneously detach from all three surfaces once the sample is immersed in water. Nevertheless, the untreated surface is still contaminated by oil, and the oil removed from the treated regions adsorbs onto other untreated areas, as shown in Fig. 6(c). After the sample is removed from the immersion bath, the two treated surfaces recover a clean state.

**Figure 7:**
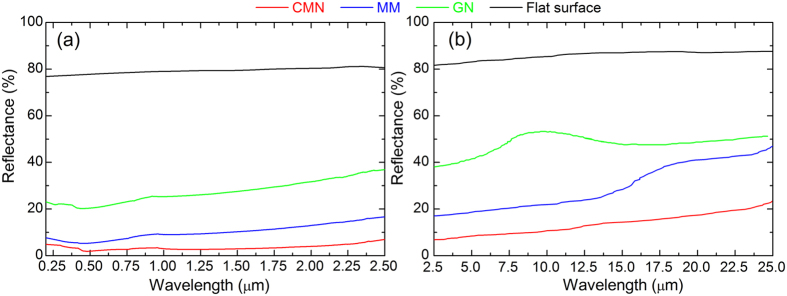
Spectral reflectance of surface structures induced by a femtosecond laser on stainless steel plates. (**a**) UV-infrared range: 0.2–2.5 μm, (**b**) mid-infrared range: 2.5–25 μm.

The spectral reflectance of the treated surfaces and the untreated flat surface over the broadband wavelength spectrum in air is also investigated. Figure 7(a) shows the spectral reflectance of the various as-prepared and control surfaces in the UV-infrared range of 0.2–2.5 μm. This figure shows that the CMN surface has the lowest reflectance (less than 8%). The reflectance of the MM surface is slightly higher than that of the CMN surface, ranging from 8% to 17%, while that of the GN surface increases to 23–37%. Compared to the polished flat surface with a reflectance of approximately 80%, all laser-treated surfaces are significantly less reflective. The spectral reflectance over a broad wavelength range of 2.5–25 μm is also investigated, as depicted in Fig. 7(b). The reflectance of the polished substrate sample without laser treatment shows a slow rising tendency from 80% to 88% with the increasing wavelength. The GN surface exhibits a large increase in reflectance at a wavelength of 5 μm, and its reflectance ranges from 37% to 52%. For the MM structures, there is a rapid increase in reflectance from 17% to 47% beginning at 15 μm. The CMN surface has the lowest reflectance (less than 25%). This result indicates that the CMN surface has strongly enhanced absorption, offering the best reflectance performance.

**Figure 8:**
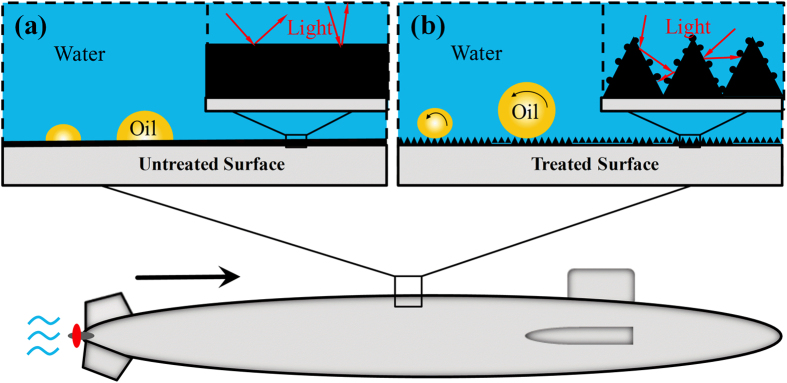
Schematic of the proposed surfaces on a submarine. (**a**) The effect of an untreated flat surface on oil-wetting and light reflectance in water. (**b**) The effect of the CMN surface on oil-wetting and light reflectance in water.

Military submarines, amphibious aircraft and tanks are commonly required to execute stealthy tasks in harsh environments, such as in oil-polluted water, and they must avoid both contamination and discovery in shallow seas or in air by radar. The metallic CMN surface provides underwater superoleophobicity, anti-oil properties and ultra-broadband enhanced absorption, thus offering a promising solution for these requirements. Figure 8(a) shows the oil-wetting and light reflectance performance of untreated flat surfaces in water. Because of the large, flat contact area between the oil and the metallic surface, oil impurities may contaminate or even corrode surfaces over time, resulting in high oil adhesion. Meanwhile, flat metallic surfaces are highly reflective because of the strong oscillation of the high free-electron density in response to external irradiation, which will in turn efficiently radiate light back to the surrounding medium^34^.

The laser-treated CMN surfaces mitigate these issues, as shown in Fig. 8b. As these surfaces are immersed in water, the water will quickly spread into the coral-like microstructures and occupy the microcavity interspace on the surface decorated with micro/nanostructures. In other words, an oil droplet on the surface will sit on the rough microstructures, and water will be trapped below the oil^35^. According to the Cassie-Baxter model, the oil droplet resides on a composite solid-water interface, forming an oil/water/solid three-phase system^35^,^36^,^37^. The water trapped at the CMN surface is a repulsive liquid phase for oil, which promotes superoleophobicity. However, the contact area between the metal surface and the oil droplet can be reduced by coral-like microstructures. As a result, the CMN surface exhibits ultralow oil adhesion. This analysis demonstrates that coral-like microstructures with nanoparticle surfaces can effectively amplify underwater superoleophobicity and anti-oil properties. Simultaneously, due to the dual effect of cavities and nanoparticles, most incident light will be trapped by these structures, resulting in a remarkable decrease in reflectance.

## Conclusions

In summary, a one-step method has been proposed to achieve underwater superoleophobicity, anti-oil properties and ultra-broadband enhanced optical absorption of metallic surfaces using femtosecond laser fabrication. Three distinct surface structures are fabricated by varying the processing parameters. All morphologies of the three surface structures, their corresponding oil wettability and reflectance spectra have been examined. The surfaces with CMN and MN demonstrate underwater superoleophobicity and anti-oil properties as well as good chemical stability with various oils and a range of pH values. In addition, the CMN surface shows strongly enhanced absorption over the broadband wavelength, ranging from 0.2–25 μm. The potential mechanism of the excellent performance of the CMN surface is also discussed. It is believed that the underwater superoleophobicity, anti-oil properties and ultra-broadband enhanced absorption of the metallic CMN surfaces have potential applications in military submarines, amphibious military aircraft and tanks and underwater anti-oil optical counter-reconnaissance devices.

## Methods

### Femtosecond laser fabrication

Stainless steel (74.0 wt.% Fe, 14.1 wt.% Cr, 10.8 wt.% Mn, 1.1 wt.% other) is a useful metallic material due to its high strength and its anti-corrosion and antioxidant properties. These properties make stainless steel an important candidate for applications such as underwater military devices and photo-electrochemical systems. In our experiments, stainless steel plates (30 mm × 20 mm × 2 mm) are polished well with abrasive paper. A schematic of the line-by-line laser scanning process experimental setup is shown in our previous work^38^. The samples are mounted on an X–Y–Z translation stage, and the laser source is an amplified Ti:sapphire laser system that generates 120 fs linearly polarized pulses at a 1 kHz repetition rate with a central wavelength of 800 nm. The Gaussian laser beam is focused on the surface of the samples with a microscope objective (10×, N.A. = 0.25) at the normal incidence. The estimated radius of the beam focus is 5 μm. A half-wave plate and a linear polarizer are used to control the laser fluence, and a mechanical shutter is used as a switch to turn the laser beam on and off. In experiments, a line-by-line scanning process is used with various laser fluences and scanning speeds, employing a fixed-move step of 10 μm between two consecutive lines. After laser irradiation, samples are rinsed for 10 min with acetone, alcohol and deionized water in an ultrasonic bath in order to clean residual material ejected from the surface.

### Characterization

The morphology of the as-prepared surface is imaged using a LMU SEM (MIRA3, TESCAN, Czech). The 3D profile of the fabricated patterns is characterized using Axio laser confocal microscopy (LSM700, Zeiss, Germany). The contact angles of a 6-μl droplet of water or 1,2-dichloroethane (C_2_H_4_Cl_2_) on the surface are measured using a contact-angle system (SL200, HARKE, China). The detailed methodology for the contact angle measurement is shown in Supplementary Fig. S4. The reflectance from 200 nm to 2.5 μm is measured using an UV-VIS-NIR spectrophotometer (Lambda 750, PE, USA). An FT-IR spectrometer (NEXUS670, Nicolet, USA) with a wavelength range of 2.5–25 μm is used to measure reflections in the mid-infrared region. Both wavelength regions are based on the integrating sphere technique.

## Additional Information

**How to cite this article**: Yin, K. *et al.* Underwater superoleophobicity, anti-oil and ultra-broadband enhanced absorption of metallic surfaces produced by a femtosecond laser inspired by fish and chameleons. *Sci. Rep.* **6**, 36557; doi: 10.1038/srep36557 (2016).

**Publisher’s note:** Springer Nature remains neutral with regard to jurisdictional claims in published maps and institutional affiliations.

## Supplementary Material

Supplementary Information
