# Supplementary material for: Underwater superoleophobicity, anti-oil and ultra-broadband enhanced absorption of metallic surfaces produced by a femtosecond laser inspired by fish and chameleons
Source: Sci Rep. 2016 Nov 7;6:36557. doi: 10.1038/srep36557 (PMC5098196; doi:10.1038/srep36557)
Supplement: Supplementary Information [file srep36557-s1.pdf]

Underwater superoleophobicity, anti-oil and ultra-broadband enhanced absorption of metallic surface produced by a femtosecond laser inspired from fish and chameleons

K. Yin, Y.X. Song, X.R. Dong, C. Wang*& J.A. Duan*

State Key Laboratory of High Performance Complex Manufacturing, School of Mechanical and Electrical Engineering, Central South University, Changsha 410083, China

Correspondence should be addressed to C.W. (email:wangcong@csu.edu.cn) or J.A.D.(email:duanjian@csu.edu.cn)

Supplementary Materials

The long term stability, the mechanical durability, and scalability of as-prepared surface

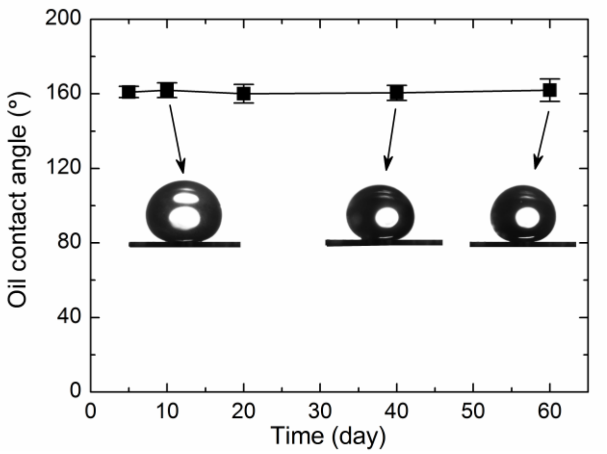

Fig. S1. Variation of oil contact angle underwater by increasing time on CMN surface

The as-prepared CMN surface is kept in closed containers filled with water for several months to examine its time durability. FigureS1 shows the curve of oil contact angle variation with respect to time. It is easily observed that there is nearly no change of the contact angle for the CMN surface, which indicates that its time durability is good and the as-prepared surface would be stable for a long time.

Moreover, the mechanical durability test for the CMN surface is carried out, as presented in Fig. S2. Firstly, friction is repeated fifty times using the lens tissue on the CMN surface. Then, a 100g weight is statically placed on the CMN surface for one day. After that, the sample is rinsed for 10 min

with acetone, alcohol and deionized water in an ultrasonic bath cleaner, respectively. The SEM image after mechanical durability test shows that these coral-like microstructures with nanoparticles are almost the same with that before the test. Meanwhile, the oil contact angle in water is about 158°, which means the underwater superoleophobicity almost has no change as before. According to the results above, it is demonstrated that the re-usability of as-prepared CMN surface is good.

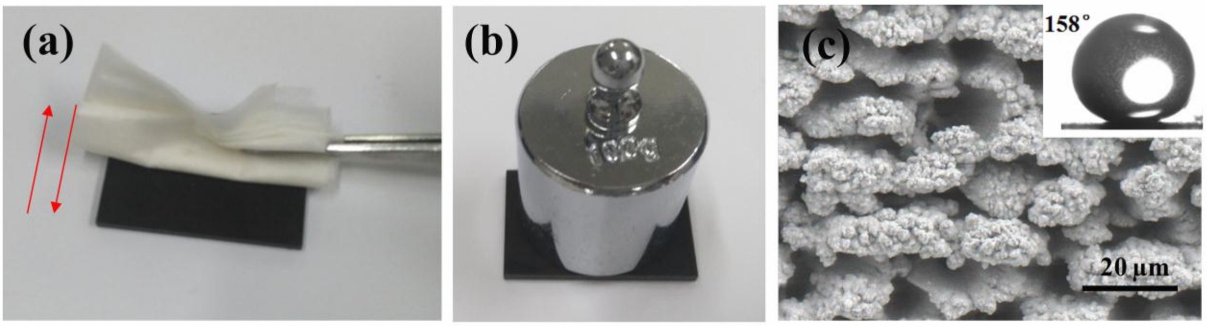

Fig. S2. The mechanical durability test for the CMN surface. (a) Repeated friction fifty times using the lens tissue. (b) Bearing 100g weights for one day. (c) SEM image of the CMN surface after mechanical durability test. The inset is the corresponding oil contact angle in water.

To gain further insight into the relationship between the droplet volume (size) and contact angle, the related experiments are performed, as shown in Fig. S3. At a relative small volume (0.5 μL and 1 μL), the oil contact angle is about 140°. As the volume increases from 3μL to 8 μL, the oil contact angles increase and the underwater superoleophobicity can be achieved. When the volume increases up to 10μL, the oil droplet has a little deformation due to the gravity and the oil contact angle has a slight decrease.

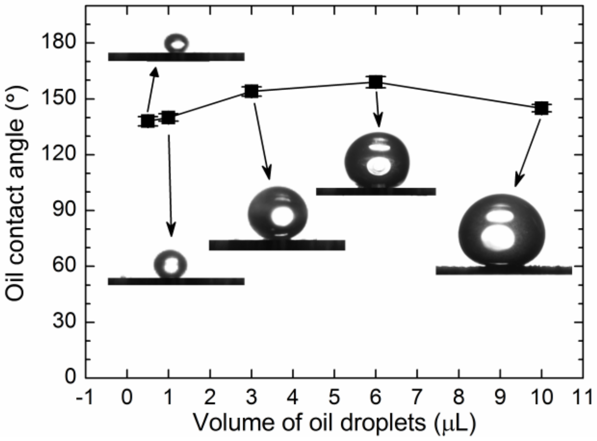

Fig. S3.Underwater oil contact angle as functions of the oil volume

The methodology for the contact angle measurement

Generally, it is extremely difficult to measure the contact angles above 150 degrees. Due to this issue, there are mainly two measurement methods [S1], as shown in Figure S4. For small droplet volume, the contact angle is assumed to form a spherical cap and is calculated by the spherical cap algorithm as depicted in Fig. S4(a), where R is the radius of curvature of the drop, d is its diameter, and θ is the mean contact angle.[S1-S3]Nevertheless, if a drop is large enough that it will have a distortion to a certain extent, in which case the spherical cap algorithm would not be applicable. More complicated method combined with the spherical cap algorithm and the tangent algorithm will be used.

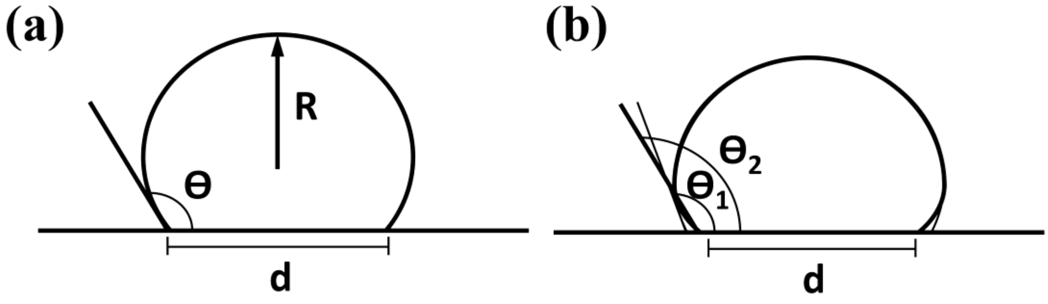

FigureS4. (a) Contact angle θ measurement using the spherical cap algorithm in the condition for the small droplet volume without droplet distortion. (b) Contact angles measured using complicated method combined with the spherical cap algorithm and the tangent algorithm in the condition for droplet deformation.

In our study, all of the oil droplets are smaller than 10 mL and there is no large oil droplet distortion. Therefore, the spherical cap algorithm is used in all of the contact angle measurement. The digital oil drop images are processed by the contact-angle system (HARKE, China), which can calculate the contact angle from the drop shape with an accuracy of ±0.1 °. In order to reduce the testing error, each contact angle is measured for five times and the average value is employed.

References

S1. Chen, F.et al. Anisotropic wetting on microstrips surface fabricated by femtosecond laser. *Langmuir* **27**, 359–365 (2010).

S2. Sheng, X. L., Zhang, J. H. & Jiang, L. Application of the restricting flow of solid edges in fabricating superhydrophobicsurfaces. *Langmuir* **25**, 9903-9907 (2009).

S3. Zhang, J. H., Gao, X. F., Jiang, L. Application of superhydrophobic edge effects in solving the liquid outflow phenomena. *Langmuir* **23**, 3230-3235 (2007).
